# Macrophage Polarity and Disease Control

**DOI:** 10.3390/ijms23010144

**Published:** 2021-12-23

**Authors:** Suguru Kadomoto, Kouji Izumi, Atsushi Mizokami

**Affiliations:** Department of Integrative Cancer Therapy and Urology, Graduate School of Medical Science, Kanazawa University, 13-1 Takaramachi, Kanazawa 920-8641, Japan; 32f3k8@bma.biglobe.ne.jp (S.K.); mizokami@staff.kanazawa-u.ac.jp (A.M.)

**Keywords:** autoimmune diseases, cell-cell interaction, microenvironment, monocytes, M1, M2, tumor-associated macrophages

## Abstract

Macrophages are present in most human tissues and have very diverse functions. Activated macrophages are usually divided into two phenotypes, M1 macrophages and M2 macrophages, which are altered by various factors such as microorganisms, tissue microenvironment, and cytokine signals. Macrophage polarity is very important for infections, inflammatory diseases, and malignancies; its management can be key in the prevention and treatment of diseases. In this review, we assess the current state of knowledge on macrophage polarity and report on its prospects as a therapeutic target.

## 1. Introduction

In 1892, microbiologist Ilya Metchnikov discovered cells that move around and eat things and termed these as macrophages [1]. There have been many studies on the origin of macrophages, and the concept of the mononuclear phagocyte system was proposed by Furth and Cohn in 1968 [2]. Monocytes are thought to emerge from bone marrow-derived precursors, drift into the circulatory system, and migrate to peripheral tissues as needed. Although this hypothesis has been believed for a long time, some tissue-indigenous macrophages have been reported to originate from the yolk sac during embryogenesis and are maintained independently of monocytes [3,4]. These facts indicate that tissue macrophages can be divided into two groups: those derived from the yolk sac during embryonic life and those derived from bone marrow precursors. However, it is still unclear what the exact functions of macrophages are, as well as if there are differences between yolk sac-derived and bone marrow-derived macrophages. In addition, some tissues have specialized macrophages, such as central nervous system microglia, bone osteoclasts, alveolar macrophages in the lungs, and Kupffer cells in the liver—all of which play an important role in maintaining tissue homeostasis [5]. Monocytes are a bone marrow-derived population that make up about 5–10% of white blood cells, and most macrophages are altered monocytes [6]. Monocytes have a lifespan of approximately two days, but this lengthens to several months when they migrate into tissues and change into macrophages, allowing them to function for a long time [6]. Macrophages are often responsible for the host defense against microorganisms by exerting immune functions, but they are also closely associated with autoimmune diseases and malignant tumors [5]. This article provides an overview of the phenotype and function of macrophages, their important role in disease pathogenesis, and implications for management.

## 2. Macrophage Polarity

Since their discovery, macrophages have long been thought to exist in only one type, but recent research findings suggest that there are multiple subtypes of macrophages [7]. As there are still many unanswered questions about macrophage subtypes, the current mainstream of research focuses on M1 and M2 macrophages [5]. The M1 and M2 of macrophages indicate their state, and depending on their localization and environment, M1 can change to M2 and vice versa [5]. Since the 2000s until today, M1 macrophages are believed to be pro-inflammatory, whereas M2 macrophages are anti-inflammatory in nature [8,9].

M1 macrophages are the most classically known macrophages, and are induced by lipopolysaccharide (LPS) and interferon (IFN)-γ and secrete pro-inflammatory cytokines such as tumor necrosis factor (TNF)-α, interleukin (IL)-1, IL-6, and inducible nitric oxide synthase (iNOS) [10]. These secretions are capable of killing infectious organisms such as bacteria, viruses, and malignant tumor cells; the dead cells are taken up by the macrophages via phagocytosis [11,12]. Any foreign substances taken up by macrophages fuse with lysosomes as vesicles and are degraded by the action of hydrolytic enzymes contained in the lysosomes [11]. Thus, M1 macrophages are thought to be involved in the maintenance of homeostasis in the human body through infection defense and anticancer effects. Excessive immune responses can lead to chronic inflammation and inflammatory diseases, and thus, the function of M1 macrophages should still be regulated [13].

Conversely, M2 macrophages are involved in tissue repair and immune tolerance [5]. M2 macrophages are induced by cytokines such as IL-4 and IL-13 via the activation of signal transducer and activator of transcription (STAT) 6 [14]. M2 macrophages secrete IL-10, arginase (ARG), and transforming growth factor (TGF)-β to suppress the inflammatory response [10]. M2 macrophages are also potent phagocytes, which act by scavenging debris and inducing both wound healing and angiogenesis [10]. Thus, M2 macrophages play a role in maintaining organs and soft tissues and regulating the immune balance. However, in studies of malignancy, tumor-associated macrophages (TAMs), which often have an M2 phenotype are known to promote tumor progression, and thus, there is a negative aspect to M2 macrophages [12].

**Figure 1:**
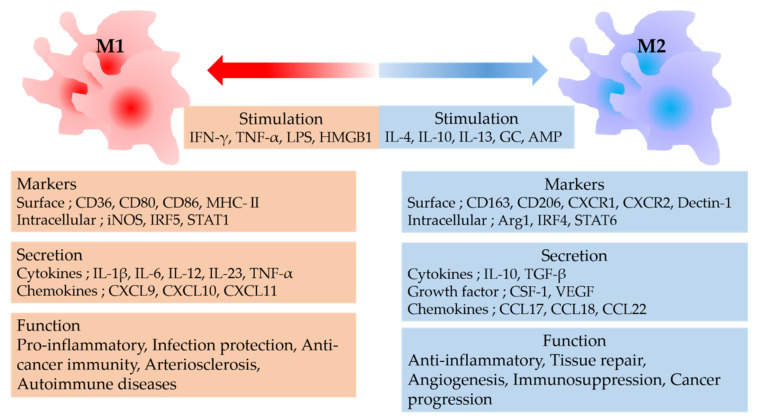
M1 macrophages are induced by IFN-γ and LPS, whereas M2 macrophages are induced by IL-4 and IL-10. M1 macrophages exert pro-inflammatory, anti-infectious, and antitumor immunity; these are responsible for inflammatory and autoimmune diseases. M2 macrophages exert anti-inflammatory effects and tissue repair but often promote tumor progression. IFN, interferon; TNF, tumor necrosis factor; LPS, lipopolysaccharide; HMGB1, high mobility group box-1 protein; IL, interleukin; GC, guanylate cyclase; AMP, adenosine monophosphate; CD, cluster of differentiation; MHC, major histocompatibility complex; NOS, nitric oxide synthase; IRF, interferon regulatory factor; STAT, signal transducer and activator of transcription; CXCR, CXC chemokine receptor; ARG, arginase; CXCL, CXC chemokine ligand; TGF, transforming growth factor; CSF-1, colony-stimulating factor-1; VEGF, vascular endothelial growth factor; CCL, CC chemokine ligand.

Thus, macrophages perform a variety of functions depending on their phenotype and are deeply involved in the maintenance of human health and in the healing or worsening of diseases (Figure 1). Therefore, it is important to manage macrophage polarity in both systemic and local treatments.

## 3. Infectious Diseases and Macrophages

Macrophages, along with other phagocytes such as neutrophils, play an important role in the host defense system by recognizing and eliminating pathogenic organisms that invade the body [15]. M1 macrophages are particularly important for infections; these are induced by LPS and IFN-γ [16]. The direct bactericidal functions of M1 macrophages include the destruction of microstructure by reactive oxygen species and phagocytosis of bacteria [16]. The indirect bactericidal action of M1 macrophages is the production of pro-inflammatory cytokines and chemokines, which recruit white blood cells and other immune cells to the site of infection [17]. In addition, M1 macrophages induce T helper 1 (Th1) type immune responses by presenting antigens to naive T cells and producing IL-12 [18]. Induction of Th1 cells into infected tissues is important because Th1 cells produce IFN-γ and enhance the phagocytic activity of M1 macrophages [18]. Thus, the M1 polarization of macrophages against invading infectious microorganisms is an important aspect of the immune system.

### 3.1. Tuberculosis and Macrophages

Tuberculosis (TB) is an infectious disease caused by the human tuberculosis bacterium *Mycobacterium* (*M*) *tuberculosis* complex, and although the lungs are the primary site of infection, it can infect bones and other organs of the body [19]. Tuberculosis is thought to affect a quarter of the world’s population and is a serious infectious disease that causes many deaths [20]. *M. tuberculosis* invades the host lung as an aerosol; this infects and destroys lung tissue. Pathogen-associated molecular patterns (PAMPs) in the cell wall of *M. tuberculosis* are recognized by pattern recognition receptors (PRRs), and macrophages are activated via these receptors [21].

Toll-like receptors (TLRs) are the most representative immune response of PRRs to infectious microorganisms, but those expressed on the surface of macrophages may not contribute much to protection against *M. tuberculosis* [21,22]. It is now known that C-type lectin receptors (CLRs) are important receptors that recognize PAMPs in *M. tuberculosis* [23]. Mincle, a member of the CLR family present in macrophages, recognizes the *M. tuberculosis* glycolipid trehalose-6,6′-dimycolate and responds by producing nitric oxide which is necessary for sterilization and induces an immune response in Th1 cells [23,24]. Dection-2 is also a CLR present in macrophages, and it is able to recognize a large number of bacteria and fungi [25]. Dection-2 recognizes the mannose-capped lipoarabinomannan of *M. tuberculosis* and exerts its bactericidal effect by stimulating the secretion of pro-inflammatory cytokines by macrophages [26]. Furthermore, TLR9 is present in macrophage phagosomes and can recognize DNA with unmethylated CpG motifs from phagocytosed bacteria [27,28]. However, some *M. tuberculosis* bacteria escape from the phagosome and move into the cytoplasm, wherein nucleotide binding-oligomerization domain (NOD) 2, a member of the NOD-like receptor family, recognizes the N-glycolyl mycobacterial DNA-binding protein (MDP) of *M. tuberculosis* [29]. NOD2 recognizing N-glycolyl MDP promotes the production of pro-inflammatory cytokines by macrophages via NF-κB activation [29].

In short, macrophages have numerous bactericidal, phagocytic, and antigen-presenting activities against *M. tuberculosis*, but *M. tuberculosis* is also equipped with various immune evasion functions. Macrophages transfer nicotinamide adenine dinucleotide phosphate (NADPH) oxidase to phagosomal membranes to generate reactive oxygen species for sterilization [30]. Phosphatidylinositol 3-phosphate (PI3P) is required for NADPH oxidase to localize to the phagosome membrane, but *M. tuberculosis* inhibits PI3P [31]. In addition, ESAT-6 secretion system 1, a type VII secretion system of *M. tuberculosis*, secretes early secreted antigenic target-6, which disrupts the membrane structure of phagosomes [32]. Thus, *M. tuberculosis* not only escapes from macrophages but also affects the macrophages themselves. Monocytes in the peripheral blood of patients with tuberculosis have been reported to exhibit M2-type properties [33]. In vitro, DnaK (from *M. tuberculosis*) was reported to induce M1 macrophages to become M2 macrophages expressing ARG and IL-10, which may suppress host immunity [34]. Taken together, these facts suggest that the management of macrophage polarity may be important in the treatment of tuberculosis and the suppression of its recurrence.

### 3.2. Human Immunodeficiency Virus (HIV) and Macrophages

HIV-1, a retrovirus, reverse transcribes its genomic RNA into DNA and incorporates it into the host chromosomal DNA. It then uses the host cell’s functions to perform viral RNA transcription and protein translation to propagate itself [35]. The emergence of potent and highly active antiretroviral therapy for HIV infection has greatly improved the lives of people infected with HIV. However, a cure is yet to be discovered, and this remains an important clinical challenge [36]. Even if the amount of HIV in the peripheral blood becomes less than the sensitivity after long-term treatment, the viral load can still increase if treatment is interrupted [37]. Thus, it has been speculated that there may be reservoirs where HIV can remain.

M1 macrophages exert antiviral functions, inhibiting viral entry and replication, as well as activating immunity in the early stages of HIV infection [38]. However, as the infection progresses, IL-10 is induced by Th2 cells to suppress inflammation, causing M2 macrophages to increase. IL-10 also increases CC chemokine receptor (CCR)5 expression in macrophages and enhances HIV entry [39,40]. Thus, M1 is predominant in the early stages of HIV infection, and M2 becomes predominant as the infection progresses [40].

HIV mainly targets cluster of differentiation (CD)4+ T cells and monocytes/macrophages using CD4 as a major receptor and CCR5 and CXC motif chemokine receptor (CXCR)4 as auxiliary receptors [41]. Therefore, macrophages are a promising reservoir candidate. In fact, HIV-1-infected macrophages have been detected in the urethra of patients with chronic HIV [42]. We can affirm that macrophages are latently infected with HIV, but it is still unclear whether this is related to the increase of the virus due to treatment interruption. Whether macrophages are true HIV reservoirs requires further analysis.

An important role of macrophages in HIV-1 infection is the transmission of the virus to CD4+ T cells. HIV-infected macrophages show increased motility [43], due to their long, thin membrane protrusions called tunneling nanotubes, which enable them to move rapidly [44]. In addition, HIV-1-infected macrophages and monocytes are involved in chronic inflammation of the blood vessels, leading to atherosclerosis [45]. HIV-1-infected monocytes can cross the blood–brain barrier and allow the virus to enter the central nervous system, causing cognitive decline in patients through direct effects on microglia and inflammatory effects on macrophages [46,47]. Thus, changes in macrophage activity associated with infection may be an important target for HIV therapy in the future.

### 3.3. COVID-19 and Macrophages

COVID-19 spread rapidly in 2020, causing a global pandemic with catastrophic effects on human society [48]. The quick development of a vaccine could prove highly effective and, with widespread use, could bring this infection under control [49,50]. However, at this time, the world has not been able to overcome this virus, and it is still difficult to determine the course of the pandemic. Viral pneumonia caused by COVID-19 can lead to severe adult respiratory distress syndrome (ARDS), which can be fatal to patients [51].

Often, in severe infections, over-activation of various immune cells leads to cytokine storm, which can lead to multiple organ failure and ARDS [52]. The cytokine storm involves pro-inflammatory cytokines such as IFN, IL-1, and IL-6; anti-inflammatory cytokines (i.e., IL-10); and various chemokines such as CC chemokine ligand (CCL)2 [53]. Macrophage activation syndrome (MAS) has been postulated as a pathology in which excessive macrophage activation causes a cytokine storm [54]. Furthermore, although most patients with COVID-19 infection are asymptomatic or have mild disease, it has been suggested that some patients with severe disease may have a MAS-induced cytokine storm [55].

MAS is a state of systemic hyperinflammation often observed in patients with rheumatic diseases such as systemic juvenile idiopathic arthritis and systemic lupus erythematosus [54]. In patients with MAS, increased serum pro-inflammatory cytokines, including TNF-α, IL-6, and IL-1β, are associated with fever, ARDS, and disseminated intravascular coagulation [56]. Patients with severe COVID-19 viral pneumonia have similar symptoms to those with MAS, specifically in the increased levels of serum cytokines [57]. Among patients with ARDS, plasma levels of TNF-α, IL-1β, IL-6, and IL-8 were higher among patients who expired versus survivors [58]. Many inflammatory cytokines are involved with M1 macrophages, and it is thought that both patients with severe COVID-19 viral pneumonia and those with MAS have an excessive inflammatory state centered on M1 macrophages [59].

These facts suggest that treatment against MAS may be effective against COVID-19 viral pneumonia. Many clinical studies are underway, focusing on IL-1β, IL-1 receptor, and IL-6 [60]. The most widely studied anti-IL-6 antibody, tocilizumab, may prevent patients from needing ventilator support, but this did not improve survival rate [61]. However, IL-1 receptor inhibitors have been reported to be effective [62]. Currently, there are no effective therapies with sufficient validation. Thus, managing macrophages and inflammatory diseases remains an important challenge.

## 4. Inflammatory Diseases and Macrophages

The inflammatory response is an important biological defense against trauma and infection. However, once inflammation occurs, it must be appropriately controlled; disruption of this mechanism can lead to chronic inflammation and tissue damage [63]. Tissues damaged by persistent inflammation range from visceral tissues to soft tissues and blood vessels [10]. Herein, we describe the relationship between representative inflammatory diseases and macrophages.

### 4.1. Atherosclerosis

Atherosclerosis is a clinically important cause of coronary artery disease and stroke [64]. In atherosclerosis, a plaque forms in the vessel wall and grows, causing stenosis and rupture associated with fatal bleeding. Therefore, prevention of atherosclerosis is a medical challenge in developed countries [64]. Atherosclerosis is inflammation caused by low-density lipoprotein (LDL) and is considered sterile due to its formation process [65]. Increased LDL in the bloodstream enters the vessel wall and is oxidized, damaging the endothelial tissue and inducing monocytes/macrophages [65]. Macrophages remove the oxidized LDL, but without a negative control mechanism, they increase in size, lose mobility, and continue to accumulate in vascular tissue, leading to plaque formation [66]. As the plaque continues to grow, macrophages become necrotic and other macrophages remove them, but they cannot keep up, leading to the formation of a necrotic core [67]. Under these conditions, inflammatory substances induce necrosis of the vascular smooth muscle, while matrix-degrading enzymes induce degradation of the fibrous membranes, leading to plaque rupture [67].

The general treatment for atherosclerosis is to reduce LDL in the blood through appropriate diet, exercise, and medication. However, macrophages could also be a therapeutic target because of their heavy involvement in plaque formation. At the site of atherosclerosis, macrophages polarize into M1 macrophages through inflammatory signaling caused by lysosomal damage associated with oxidized LDL phagocytosis and TLR4 signaling activation by oxidized LDL [68,69]. Macrophages produce IL-1β, a pro-inflammatory cytokine. Canakinumab, an anti-IL-1β antibody, was found to reduce adverse cardiovascular events, but affected host immunity and increased the risk of severe infections [70]. Plaque control by suppressing inflammation via regulatory T cells (Treg) has also been attempted [71]. In summary, the advancements in the treatment of atherosclerosis include managing the vessel wall through the regulation of inflammation by immune cells such as macrophages.

### 4.2. Obesity

Obesity is closely associated with hypertension, type 2 diabetes, and malignancies and is a major healthcare concern in developed countries [72]. Obesity-induced adipose tissue growth increases iNOS and chemokines, which recruit monocytes/macrophages and induce them to become inflammatory M1 macrophages [6]. In turn, the increased number of M1 macrophages releases IL-1β and IL-6, which counteract the insulin-sensitizing effects of adiponectin and leptin, thus inducing insulin resistance [73]. Thus, obese people are more polarized toward M1 macrophages, whereas thin people are more polarized toward M2 macrophages that produce IL-10 and Arg-1 [74,75].

M2 macrophages are thought to be involved in maintaining adipose tissue homeostasis, preventing inflammation, and promoting insulin sensitivity [74,75]. However, M2 macrophages produce IL-10, an anti-inflammatory cytokine. IL-10-deficient mice were found to have increased insulin sensitivity and improved glucose tolerance [76]. Furthermore, decreasing the expression of the IL-10 receptor (IL-10R)α in adipocytes would enhance metabolism. These results indicate that M2 macrophage-derived IL-10 suppresses fat metabolism by acting on IL-10Rα [76]. M2 macrophages inhibit adipocyte growth by inhibiting proliferation of adipocyte progenitors [77].

Removal of M2 macrophages promotes the proliferation of small adipocytes and increased glucose uptake, resulting in lower blood glucose levels [77].

Thus, M2 macrophages are often good for fat metabolism, but not always so for glycemic control, which is often associated with obesity, and may be a new target for diabetes treatment.

## 5. Cancer and Macrophages

In cancer tissue, there are numerous immune cells, fibroblasts, and epithelial cells, which comprise the tumor microenvironment (TME) and are closely involved in the growth and progression of cancer cells [78]. Cancer cells secrete monocyte chemotactic factors (mainly CCL2) to recruit monocytes/macrophages to the TME [29]. In many cases, macrophages express M1-type markers and exert anticancer effects with other immune cells, but in the TME, they are in an unusual state [79]. These macrophages are called TAMs, which secrete angiogenic and immunosuppressive factors, as well as promote tumor growth, invasion, and metastasis through tissue destruction and remodeling [79].

Macrophages are pluripotent immune cells and therefore secrete a large number of cytokines [29]. TAMs often express M2 macrophage markers such as CD163 and CD206 [80]. Recently, however, some TAMs were found to express both M1 and M2 markers. M1-type TAMs were even found to contribute to tumor progression, so it is no longer correct to assume that TAMs are always M2 macrophages [81]. TAMs suppress antitumor immunity as well as promote angiogenesis, tumor growth, tumor invasion, and metastasis [79,82].

### 5.1. Cancer Progression Mechanism of TAMs

Cancer cells secrete a variety of factors that induce TAMs by activating AKT/mTOR and ERK/STAT3 signaling [83,84]. TAMs secrete cell growth factors such as TNF-α, TGF-β, epidermal growth factor (EGF), and platelet-derived growth factor, which induce cancer tissue growth [79,82]. Activation of EGF/STAT3 signaling and TNF-α/nuclear factor-kappa B signaling by TAMs promotes tumor growth and progression [85]. Angiogenesis is a very important process in supplying nutrients to tumors and creating pathways for metastasis [86]. TAMs secrete a variety of angiogenic factors, especially vascular endothelial growth factor, which has a strong effect and is a therapeutic target in many cancers [86,87].

Among the different cytokines, chemokines are leukocyte chemotactic factors of relatively small molecular weight, but they are also important in TME [88]. Inhibition of CCL2 is important because CCL2 produced by cancer cells recruits macrophages to the TME and directly increases the metastatic potential of cancer cells [89,90]. The regulation of chemokines in TME is important because TAMs also produce chemokines that act on cancer cells, immune cells, and stromal cells [91,92].

In recent years, the focus of drug therapy for cancers such as lung cancer, kidney cancer, and melanoma has shifted to immune checkpoint inhibitors [93,94,95]. Most immune checkpoint inhibitors target the programmed death receptor-1-programmed cell death ligand-1 (PD-L1) axis. However, TAMs are also becoming increasingly important in cancer therapy; these suppress cytotoxic T cells by secreting PD-L1 and induce Treg by secreting IL-10 [96]. Furthermore, TAMs may maintain tumor immunosuppressive capacity by increasing PD-L2 secretion when PD-L1 is suppressed [97]. CD25, which has a high affinity for IL-2, is abundantly expressed on the surface of Treg. When CD25 is consumed by IL-2, it inhibits the activation of antigen-presenting cells [98].

They decrease anti-tumor immunity by suppressing cytotoxic T cells and natural killer cells by secreting bone marrow-derived suppressor cells (MDSCs), ARG-1, iNOS, and IL-10 [99].

Two main types of MDSCs have been reported: monocyte-like and granulocyte-like [99]. Monocyte-like MDSCs can be recruited to cancer tissues by chemokines and can also be converted to TAMs in hypoxic environments, thus MDSCs are a source of TAMs [99,100]. Thus, TAMs play a central function in TME, and their regulation and suppression are very important in cancer therapy.

### 5.2. Regulation and Cancer Therapy for TAMs

It is clear from previous reports that TAMs suppress antitumor immunity and promote tumor growth and progression. Therefore, many cancer therapies targeting TAMs have been investigated (Table 1). One of the targets of TAM control is the CCL2–CCR2 axis [91]. CCL2 secreted by cancer cells is a typical monocyte/macrophage chemotactic factor that strongly recruits macrophages to the TME [90]. Therefore, inhibition of the CCL2–CCR2 axis may reduce the supply of TAMs to the TME. CCL2 may also be an important therapeutic target because it acts directly on tumor cells to promote tumor growth, progression, and resistance to chemotherapy [89]. Propagermanium, a drug for chronic hepatitis B, acts by inhibiting CCL2, and it is currently being studied for use in breast cancer [101]. Blocking antibodies against CCL2 have been clinically studied in prostate cancer, but inhibition of the CCL2–CCR2 axis leads to a decrease in monocytes and an increase in CCL2 levels rather than a therapeutic response [102]. Meanwhile, the CCR2 inhibitor PF-04136309 was used in combination with chemotherapy for pancreatic cancer but did not show sufficient efficacy [103]. Currently, a drug (BMS-813160) that is expected to suppress both CCR2 and CCR5 and inhibit the mobilization of TAMs to the TME is also under clinical investigation (NCT03767582).

Colony-stimulating factor 1 receptor (CSF-1R) is a very important factor in macrophage differentiation [104]. CSF-1 and IL-34 have been identified as CSF-1R ligands, and they are also potential targets for therapy [105]. Many drugs targeting CSF-1R have been developed and are currently under clinical investigation [106]. Pexidartinib has demonstrated good therapeutic efficacy as a single agent in tenosynovial giant cell tumors [107]. However, the therapeutic effect of CSF-1R inhibitors alone may be inadequate, and the mobilization of MDSCs into TME as a compensatory effect has been suggested as a possible cause [108]. For this reason, combination therapy of CSF-1R inhibitors with other anticancer agents and immune checkpoint inhibitors is being studied.

As another approach to TAM regulation, CD47, which signals to avoid phagocytosis from macrophages, could be a target [109]. In fact, anti-CD47 antibodies, in combination with anti-CD20 antibodies, have shown good therapeutic efficacy against non-Hodgkin’s lymphoma [110]. Trabectedin has also been reported to induce caspase-8-mediated apoptosis in TAMs, whereas zoledronic acid inhibits TAM differentiation [111,112]. Macrophages are becoming increasingly important in cancer therapy, and research into their regulation is ongoing.

## 6. Conclusions

**Figure 2:**
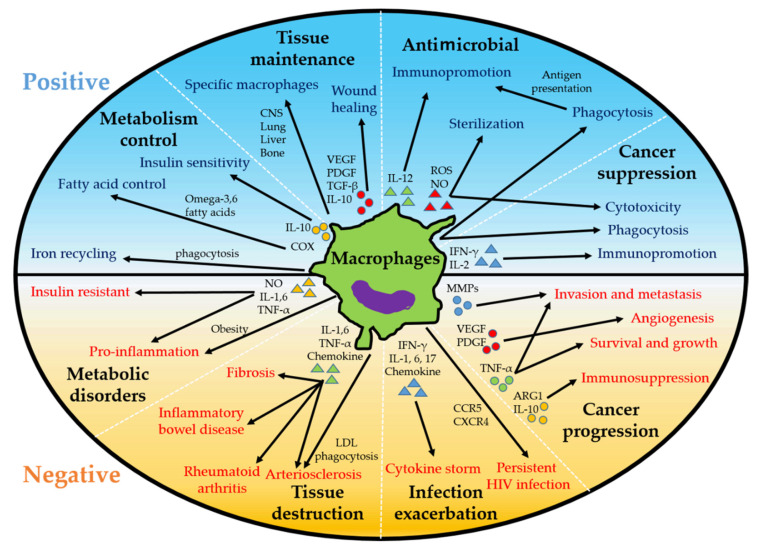
Positive and negative roles of macrophages. Macrophages exert antitumor effects but can act to promote tumor progression. Macrophages exert antimicrobial effects but can be targets of viruses and cause cytokine storms. Macrophages are responsible for tissue repair and metabolic control but can also cause tissue destruction and metabolic disturbances due to inflammation. IFN, interferon; IL, interleukin; ROS, reactive oxygen species; NO, nitric oxide; VEGF, vascular endothelial growth factor; PDGF, platelet-derived growth factor; COX, cyclooxygenase; MMPs, matrix metalloprotease; TNF, tumor necrosis factor; ARG, arginase; CCR, CC chemokine receptor; CXCR, CXC chemokine receptor; LDL, low-density lipoprotein.

Macrophages are widely distributed in the human body and exert various functions to influence a wide range of diseases such as infectious diseases, malignant tumors, and inflammatory diseases (Figure 2). Therefore, it is becoming increasingly important to understand the state and polarity of macrophages, as their regulation can be a therapeutic target. Specifically, it may enable survival from COVID-19 and HIV viruses, suppression of inflammation, and inhibition of tumor progression. Various indications for targeted therapeutic agents against macrophages are still being investigated, but none of them can completely control macrophages. In other words, there are many factors that affect macrophages, and it may be very difficult to control them with a few factors. Therefore, further research in this field, including macrophages as well as other immune cells, is important.

## Figures and Tables

**Table 1:** Drugs targeting TAMs.

Drug	Drug Type	Target Factor	Clinical Trial Number	Tumor
PF-04136309	CCR2 inhibitor	CCL2–CCR2	NCT02732938 NCT01413022	PDAC PDAC
CNTO 888	CCL2 antibody	CCL2–CCR2	NCT00992186	CRPC
BMS-813160	CCR2/5 inhibitor	CCL2–CCR2/5	NCT03184870 NCT03496662 NCT03767582 NCT04123379	PDAC, CRC PDAC PDAC NSCLC, HCC
PLX-3397	CSF-1R inhibitor	CSF-1R	NCT02452424 NCT02777710	Melanoma, NSCLC, etc. PDAC, CRC
RG-7155	CSF-1R inhibitor	CSF-1R	NCT02323191 NCT01494688 NCT02760797	Melanoma, TNBC, etc. TNBC, CRC, etc. TNBC, CRC, etc.
AMG-820	CSF-1R inhibitor	CSF-1R	NCT02713529	PDAC, NSCLC
BMS-986253	CXCL8 antibody	CXCL8–CXCR1/2	NCT03689699 NCT04050462 NCT04123379	HSPC HCC NSCLC, HCC
AZD5069	CXCR2 antagonist	CXCL8–CXCR1/2	NCT03177187	CRPC
RO7009789	CD40 agonist	CD40	NCT02588443	PDAC
Hu5F9-G4	CD47 antibody	CD47	NCT02953509	Non-Hodgkin’s Lymphoma
IPI-549	PI3Kγ inhibitor	PI3Kγ	NCT03961698 NCT02637531	TNBC, RCC NSCLC, TNBC, etc.
TMP195	Class IIa HDAC inhibitor	Class IIa HDAC	None	None
Trabectedin	small molecule	Caspase 8	None	None
Zoledronate	small molecule	NA	None	one

CCR, CC chemokine receptor; CCL, CC chemokine ligand; PDAC, pancreatic ductal adenocarcinoma; CRPC, castration-resistant prostate cancer; CRC, colorectal cancer; NSCLC, non-small cell lung cancer; HCC, hepatocellular carcinoma; CSF-1R, colony-stimulating factor-1 receptor; CXCL, CXC chemokine ligand; CXCR, CXC chemokine receptor; CD, cluster of differentiation; PI3k, phosphoinositide 3-kinase; TNBC, triple-negative breast cancer; HDAC, histone deacetylase.
